# Study on the Classification of Metal Objects by a Fluxgate Magnetometer Cube Structure

**DOI:** 10.3390/s22197653

**Published:** 2022-10-09

**Authors:** Songtong Han, Bo Zhang, Zhu Wen, Chunwei Zhang, Yong He

**Affiliations:** 1 School of Mechanical Engineering, Nanjing University of Science and Technology, Nanjing 210094, China; 2 State Key Laboratory of Disaster Prevention and Mitigation of Explosion and Impact, The Army Engineering University of PLA, Nanjing 210094, China; 3 Department of Public Security, Sichuan Police College, Luzhou 646000, China

**Keywords:** magnetic targets, ResNet-18, classification, magnetic dipole, cluster analysis

## Abstract

After wars, some unexploded bombs remained underground, and these faulty bombs seriously threaten the safety of people. The ability to accurately identify targets is crucial for subsequent mining work. A deep learning algorithm is used to recognize targets, which significantly improves recognition accuracy compared with the traditional recognition algorithm for measuring the magnetic moment of the target and the included geomagnetism angle. In this paper, a ResNet-18-based recognition system is presented for classifying metallic object types. First, a fluxgate magnetometer cube arrangement structure (FMCAS) magnetic field feature collector is constructed, utilizing an eight-fluxgate magnetometer sensor array structure that provides a 400 mm separation between each sensitive unit. Magnetic field data are acquired, along an east–west survey line on the northern side of the measured target using the FMCAS. Next, the location and type of targets are modified to create a database of magnetic target models, increasing the diversity of the training dataset. The experimental dataset is constructed by constructing the magnetic flux density tensor matrix. Finally, the enhanced ResNet-18 is used to train the data for the classification recognition recognizer. According to the test findings of 107 validation set groups, this method’s recognition accuracy is 84.1 percent. With a recognition accuracy rate of 96.3 percent, a recall rate of 96.4 percent, and a precision rate of 96.4 percent, the target with the largest magnetic moment has the best recognition impact. Experimental findings demonstrate that our enhanced RestNet-18 network can efficiently classify metallic items. This provides a new idea for underground metal target identification and classification.

## 1. Introduction

In our daily life, the geomagnetic field is always around us. The Earth’s magnetic field has its sources inside the Earth (internal contributions) and outside it (external contributions). The predominant internal source is the core field (also called “main field”), originating in the external fluid core, and the lithospheric field (also called “crustal field”), caused by magnetic minerals in the crust and, to a lesser extent, the upper mantle. The external sources originate in the ionosphere, the magnetosphere, and also from electrical currents coupling the ionosphere and magnetosphere (named “field-aligned currents”, or FAC). These external sources induce secondary fields in the Earth [1].

Maxwell established Maxwell’s equations, which essentially revealed the essential relationship between electricity and magnetism. Khan et al. [2] investigated the Hall current effect, entropy generation, Arrhenius activation energy, and binary chemical reactions in Maxwell nanofluid heat and mass transfer biological convection. Khan et al. exposed the deeper meaning of Maxwell’s equations. When a metal target (mostly iron, cobalt, nickel, etc.) infiltrates the geomagnetic field, the target is magnetized by the geomagnetic field, and a secondary field is formed in the space of the original magnetic field, distorting the surrounding magnetic field [3]. Detecting the magnetic field in space to identify subsurface [4,5] or underwater metal targets [6] has several applications in the mine detection field [7].

There are many technical techniques for detecting metal targets, with magnetic detection being one of the most prevalent. Detecting the total magnetic field, the vector magnetic field, the gradient magnetic field, and the gradient tensor magnetic field are all popular methods for measuring magnetic fields in space [7]. Different techniques for detecting the space magnetic field result in varying numbers of sensors, and sensors with a sensible arrangement collect more information about the space magnetic field [8]. In recent years, several researchers [9,10] have embraced the technical methods of multisensor collaboration for metal target identification and localization studies.

Metal targets can have a variety of exterior forms. When the target is distant from the observing location, it can be compared to a magnetic dipole model [11]. Jin et al. [12] advocated using the equivalent approach of the simplest dipole model to replace the target, which significantly simplifies the identification and inversion of metal targets. Nara et al. [13] presented a simple algorithm and a compact sensor for the localization of a magnetic dipole. They developed a sensor unit consisting of three orthogonal loop coils, three orthogonal planar gradiometers, and three orthogonal coaxial gradiometers, for measuring the magnetic field, nondiagonal, and diagonal components of the gradient tensor, respectively. Localization experiments were conducted, where the maximum error is about 7 mm when the source-sensor distance is from 80 to 140 mm. Kasatkin et al. [14] studied the solution uniqueness analysis of the magnetic dipole location problem based on the two-point known magnetic field intensity vector value. It was shown that the usage of two triples (pairs) of sensors is sufficient to solve a 3D (2D) problem of arbitrary magnetic dipole localization with a satisfactory number of crude errors. Birsan [15] proposed a recursive method, which is expected to use the data collected by the gradiometer to estimate the trajectory of the target and the magnetic moment component of the magnetic dipole source model. In his study, the determination of target position, magnetic moment, and velocity are formulated as a Bayesian estimation problem for dynamic systems, which could be solved using a sequential Monte Carlo-based approach known as the “particle filter”. Fan et al. [16] presented a fast linear algorithm for locating the target based on the total magnetic field gradient. Compared with the optimization algorithms, the proposed method provides good performance within a short time and can be used to locate the target in real time. Based on the cube tensor measurement array from the STAR method, Liu et al. [17] proposed a novel way to calculate the distance between the dipole and the sensor array. Results of simulation experiments indicate that this method increases the successful localization area by 43% compared with the traditional method, and the baseline length of the sensor array dominates the performance of this method. Yin et al. [18] presented a simple formula for the localization of a magnetic dipole. Numerical simulations show that the proposed localization formula is correct and it can also localize the magnetic dipole precisely for applications with measurement noise.

Billings et al. [19] considered the metal target a magnetic dipole and determined the detection target by studying the dipole’s amplitude and direction. Oruç [20] saw the metal target as a dipole and utilized tensor invariants to perform tensor analysis to find metal targets. In metal target location and identification research, these scholars identified the position of metal targets using different algorithms. Nevertheless, it is difficult to identify the metal target types due to algorithm limits.

Metal object recognition is a nonlinear problem that is challenging to solve. Model outputs from one location at a site were used to train a PNN model, which could correctly discriminate UXO from scrap at a different location of the same site [21]. Through careful selection of the probability threshold cutoff, the UXO detection rate obtained was 95% with a false alarm rate of only 37%. The ability to distinguish individual UXO types has been demonstrated with correct classifications between 71% and 95%. The GEM-3 sensor is a frequency-domain sensor with up to ten frequencies available for simultaneous measurement of the in-phase and quadrature response of the target. Working around a modified GEM-3 sensor, Nelson et al. [22] designed a three-sensor array and demonstrated it at the Standardized UXO Demonstration sites at Aberdeen Proving Ground and Yuma Proving Ground. Shamatava et al. [23] presented the inversion and classification performance of the advanced electromagnetic induction inversion, processing, and discrimination schemes developed by the group when applied to the Environmental Security Technology Certification Program Live-Site UXO Discrimination Study carried out at the former Camp Butner in North Carolina. Bijamov et al. [24] demonstrated in detail a semisupervised scheme to classify UXO by using as an example the data collected with a time-domain electromagnetic towed array detection system during a live-site blind test conducted at the former Camp Butner in North Carolina, USA.

Billings et al. [25] claimed that the kind of metal target might be determined by measuring the angle between the target’s remanent magnetization and geomagnetism. This approach is highly efficient, but its resolution is subpar. The metal target was derived by Beran et al. [26] by solving the goal function. Due to the nonlinear nature of the forward model, the outcome of calculating the loss function is a local minimum. Wigh et al. [27] proposed a probabilistic approach for inferring metal objects from magnetic data. Various iterative methods are utilized, as well as the multichain Markov chain Monte Carlo (MCMC) method. The method is unaffected by the starting point of the inversion process, allowing it to avoid several local minima in the highly nonlinear model space and iteration. This technique does not, however, prevent the requirement to select a beginning value. When there is no previous condition, it will be far more difficult to repeat.

Zhou et al. [28] proposed a region convolutional neural network-based (RCNN-based) method for shallow magnetic target detection and classification in metal target recognition. Zhou et al. tenderized the magnetic field to obtain the magnetic gradient tensor G in space. The G_zz_ represents the gradient of the z-direction magnetic field along the z-direction. Zhou et al. stated in the article that the z-axis points to the ground. The G_zz_ component of the magnetic gradient tensor was selected as the data source during the study procedure. To identify the target, a mask region convolutional neural network (Mask-RCNN) algorithm was developed. The study approach employs L-shaped, concave, spherical, and cuboid detection targets. However, ambient magnetic field fluctuations are not factored into the classification procedure. Therefore, the research conclusions are still theoretical. In addition, the magnetic properties of the target are insufficient since Zhou et al. only used the G_zz_ component to extract characteristics in their research.

Based on those mentioned above, this study proposed a technique for classifying metal products based on the residual neural network 18 (ResNet-18) deep learning model. Using the magnetic field data of a survey line, we accurately identify the kind of subsurface metal targets by extracting the eight fluxgate magnetometer data on the north side of the target.

## 2. Methods

### 2.1. Space Magnetic Flux Density Acquisition

When the metal target is far from the observation point, the magnetization field model of the metal target is nearly comparable to the magnetic dipole model [29]. The distribution of the magnetic field of a magnetic dipole in space may be described by the following formula [30]:

(1) $$B_{dipolar}\left(r\right)=\frac{\mu_{0}}{4\pi }\frac{1}{r^{3}}\left[3\left(m\cdot \hat{r}\right)\hat{r}-m\right],$$

where ***m*** is the magnetic moment of the dipole [L^2^I], *r* denotes the distance between the magnetic dipole’s center and the observation point, $\hat{r}$ denotes the unit vector between the magnetic dipole’s center and the observation point, and *μ*_0_ denotes the vacuum permeability.

**Figure 1:**
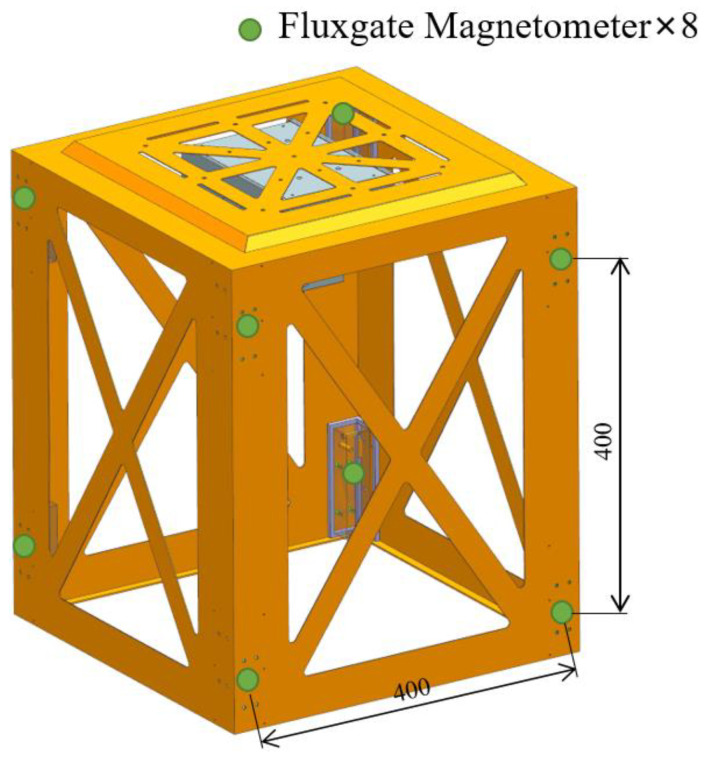
FMCAS diagram.

To obtain more data on spatial magnetic anomalies and ensure that the distance between each fluxgate sensitive unit is 400 mm, we adopted an array structure arrangement of 8 three-axis fluxgate magnetometers (model HSF923-2H5-AA Xi’an Huashun). The performance parameters of the fluxgate magnetometer are shown in Table 1. The fluxgate magnetometer has three sensitive units corresponding to three vertical magnetic fields, and the measurement range of each sensitive unit is −100,000 nT to 100,000 nT. We carried out experiments in Nanjing, where the local geomagnetic field is 50,000 nT ± 1000 nT. After placing the metal target, the maximum magnetic anomaly in the area does not exceed 60,000 nT. This type of fluxgate magnetic magnetometer can correctly estimate the magnetic field of the experimental area during the experiment. We call this structure the fluxgate magnetometer cube arrangement structure (FMCAS), as shown in Figure 1. Each fluxgate magnetometer obtains *xyz* three-axis magnetic flux data, so FMCAS obtains 8 *xyz* three-axis magnetic flux data, for a total of 24 groups of data.

**Figure 2:**
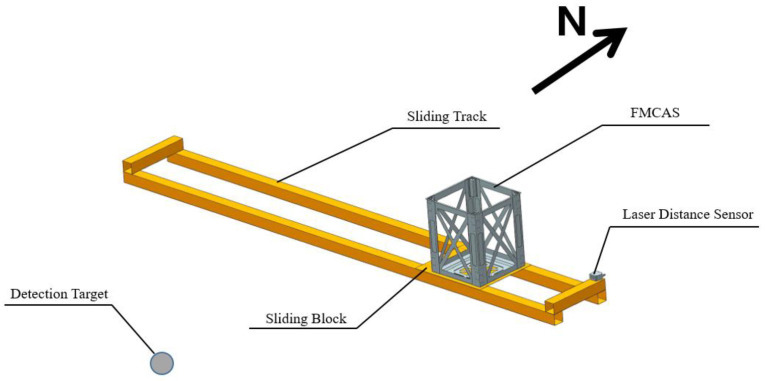
Schematic diagram of measuring line magnetic flux density acquisition.

We gather an east–west magnetic field data measurement line north of the detection target. As illustrated in Figure 2, we attach the FMCAS to the sliding block with copper bolts and position it on an aluminum-alloy sliding track. The laser distance sensor (type L-GAGE) captures information on the distance between the FMCAS and the sensor. The material of the sliding block is wood; we put oil on the sliding track to promote sliding. We obtain experimental data on a measuring line every time the assistant pulls the sliding track with the rope.

Through the above experimental method, we can obtain the magnetic field data of a survey line every time we slide the sliding block, which contains the target magnetic anomaly. We intercept the middle part of the survey line (the data at both ends fluctuate greatly when sliding starts and ends), divide the intercepted part of the survey line into 100 equal parts, and obtain 101 position coordinates. Corresponding FMCAS magnetic flux density data for each position obtain a set of 101 × 24 size magnetic flux data about the position information.

We might also define the pseudo total field *xyz*_i_ of the fluxgate magnetometer (because the fluxgate magnetometer has not been calibrated) as follows:

(2) $$xyz_{i}=\sqrt{x_{i}{}^{2}+y_{i}{}^{2}+z_{i}{}^{2}}\left(i=1,2,\cdot \cdot \cdot ,8\right)$$

The pseudo total field of the ith fluxgate magnetometer is represented by *xyz*_i_ in the formula. The *i*th fluxgate magnetometer’s x-axis magnetic field output is *x*_i_, the ith fluxgate magnetometer’s y-axis magnetic field output is *y*_i_, and the *i*th fluxgate magnetometer’s z-axis magnetic field output is *z_i_*.

**Figure 3:**
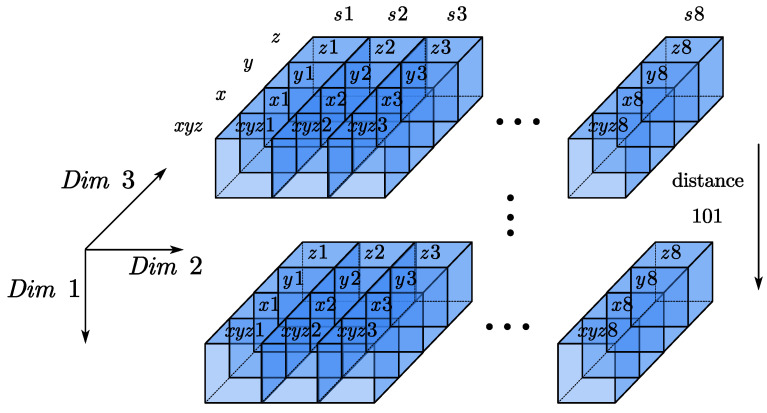
Schematic diagram of the magnetic flux tensor matrix structure.

We construct the magnetic flux tensor matrix as shown in Figure 3; the matrix size is [101, 8, 4]. The first dimension includes the 101 position information, the second dimension includes the label information of 8 fluxgate magnetometers, the third dimension includes the *xyz* pseudo total field, the x-axis magnetic field, the y-axis magnetic field, and the component information of the z-axis magnetic field.

Later, we train the recognition algorithm using the magnetic flux tensor matrix.

### 2.2. ResNet-18

Four Chinese researchers, including Microsoft Research Institute’s Kaiming He, proposed the residual neural network (ResNet). With an error rate of 3.57% on the top 5, a 152-layer neural network was successfully trained and won the championship in the ILSVRC2015 competition utilizing the ResNet unit. ResNet’s topology allows for rapid neural network training while considerably improving model accuracy.

As the neural network is expanded, the gradient explosion and disappearance problem arises. In addition, we employ normalized initialization [31,32,33] and intermediate normalization layers [34] to address this issue. Each input-to-output process is almost irreversible (information loss) due to the existence of the nonlinear ReLU activation function [35]. It is difficult to reverse the whole input from the output, which makes it highly unlikely that the features will be fully retained throughout layer-by-layer forward propagation. The residual learning module is incorporated into the deep neural network, the output before the previous layer (or layers) is added to the output calculated by this layer by jumping, and the summation result is input into the activation function as a function of the output of this layer [36]. Thus, the network’s depth can be significantly expanded. Table 2 depicts the principal ResNet structure.

We research object classification and recognition, utilizing the main network architecture of ResNet-18. In practice, we make the following modifications to the original ResNet-18:

Step 1. Remove the first layer of the 7 × 7 convolution in the original network.

Step 2. Modify the original second 3 × 3 maximum pooling layer to a 3 × 3 convolution and set 64 convolution kernels.

Step 3. Finally, the 1000 neurons in the final fully connected layer are modified to 3 neurons.

**Figure 4:**
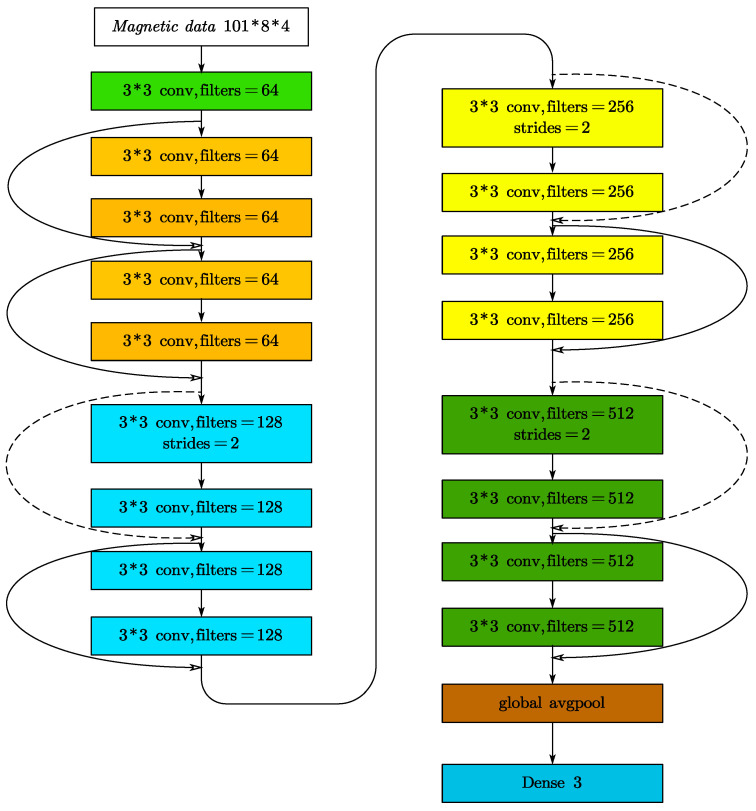
ResNet-18 network structure. The dotted line indicates that the dimensions are different, and it needs to be adjusted through a 1 * 1 convolution kernel to adjust to the same dimension.

Through the above processing, we obtain an improved ResNet-18 network. The magnetic feature data are in the data format of 101 × 8 × 4, and the convolution operation is performed through a 3 × 3 convolution kernel. Then, they are input into 8 ResNet blocks, and the corresponding convolution operation is performed. The data are then globally pooled before being fed into the fully connected layer. Figure 4 depicts the network structure. 

## 3. Training ResNet-18 Model

All model training and test evaluation experiments are carried out on an NVIDIA GeForce RTX 2080 Ti GPU with 32 GB of memory. The TensorFlow GPU version operates on Windows 10 and is installed on the Anaconda 3 platform.

### 3.1. Dataset Generation

**Figure 5:**
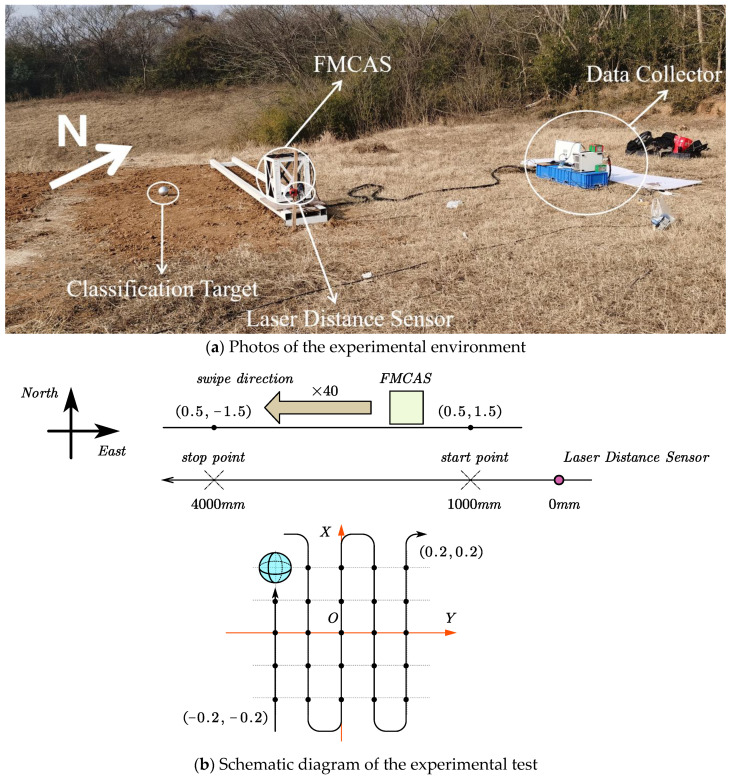
Experimental site map and experimental schematic.

In the experiment, we use three sizes of iron balls as neural network training and recognition targets. The 5 m-long sliding track is located on the north side of the detection target, and the laser distance sensor used to record the FMCAS position information is located on the east side of the sliding track. The data collector (model: PXIe-4309-NI) is located 4 m northeast of the slide rail, as shown in Figure 5a.

A spatial Cartesian coordinate system is developed, as shown in Figure 5b, with the x-axis facing north, the y-axis facing west, and the unit is meters. The start of the sliding track is at coordinates (0.5, −1.5), and the end of the sliding track is at coordinates (0.5, 1.5). The FMSC slides from the sliding track start point to the sliding track endpoint. The sliding distance is 4 m. During the sliding process, the laser distance sensor records the distance (0~4.5) of the FMSC relative to the starting point of the slide rail in real time.

The starting position of each probe target is (−0.2, −0.2), and the end position is (0.2, 0.2). The target moves 0.1 m along the x-axis each time. When the x-axis coordinate of the target reaches 0.2 m, the target moves 0.1 m along the y-axis. The x-axis position of the target is repeatedly adjusted to complete a cycle. The target has a total of 25 position points; for each position point, FMCAS slides 40 times. Each target to be tested has a total of 1000 groups of data, which are used as the training set for deep learning. The targets are randomly arranged in 5 random positions within a square area from (−0.2, −0.2) to (0.2, 0.2), and the FMCAS is also slid 40 times, with a total of 200 groups of data for each target, as the test set of deep learning. The three targets we measured and their effective data during the experiment are shown in Table 3. During the follow-up experimental processing, we found a set of experiments where the laser distance sensor did not capture the entire sliding distance of the FMCAS. Therefore, we consider the shuffling experimental data invalid, which cannot be used for subsequent identification work.

**Figure 6:**
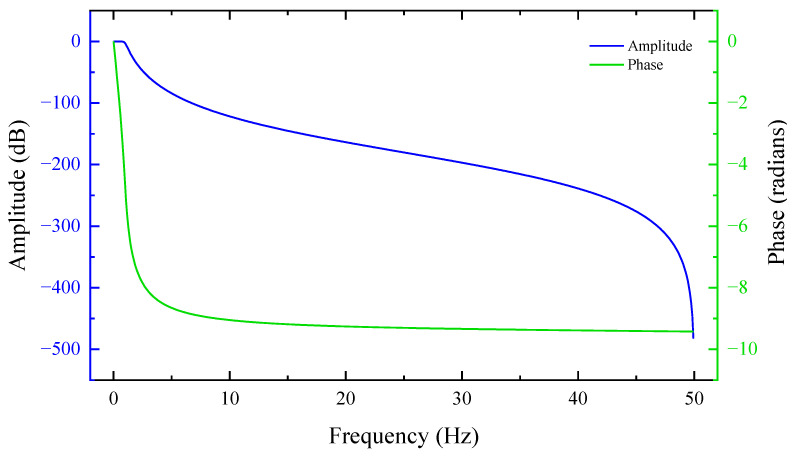
Butterworth Low-Pass Filter Frequency Response and Phase Frequency Curves.

**Figure 7:**
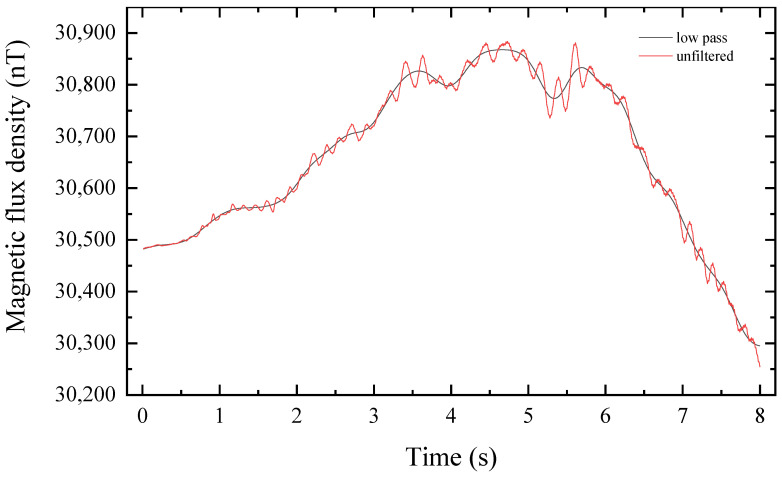
Comparison of time-domain signals before and after filtering.

We use three different sizes of shot balls as detection targets. The lead ball is cast from cast iron. The susceptibility of the material is greater than 200. Throughout the experiment, the sampling frequency is set to 100 Hz. The power frequency signal perturbs the region where the experiment is conducted, and the DC component is the magnetic anomaly generated by the target. Therefore, the collected time-domain signal is low-pass filtered using the Butterworth filter in signal processing. The bandpass frequency is set to 2 Hz, the bandstop frequency is set to 12 Hz, and the filter is set to the 6th order. Figure 6 illustrates the low-pass filter’s performance. Figure 7 depicts the comparison before and after low-pass filtering. The red curve represents the unfiltered time-domain signal, whereas the black line represents the time-domain signal that was low-pass filtered.

**Figure 8:**
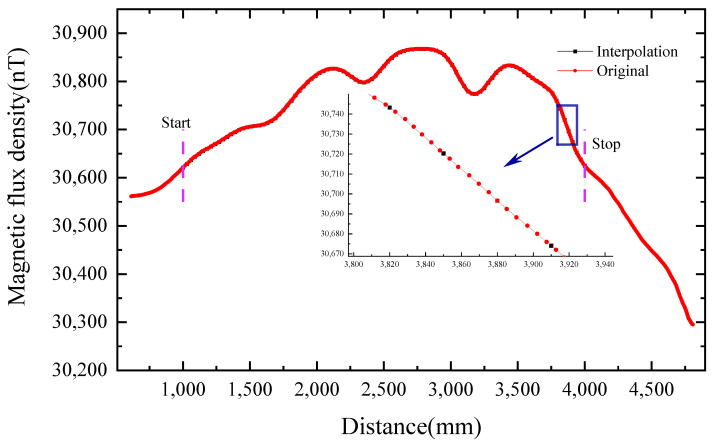
Interpolation calculation of the magnetic field position distribution.

We place the starting point of the magnetic tensor matrix at 1000 mm from the FMCAS to the laser distance sensor and the endpoint at 4000 mm. We divide 101 points evenly within a distance of 3000 mm and obtain the three-axis magnetic flux density output of each fluxgate magnetic sensor at these distance points. We use the position of the FMCAS as the independent variable and use the three-axis magnetic flux density of each fluxgate magnetic sensor as the dependent variable for the interpolation calculation. For interpolation calculations, we use the position of the FMCAS as the independent variable and the three-axis magnetic flux density of each fluxgate magnetic sensor as the dependent variable. Using the linear interpolation method of sample points, according to the position we need, the three-axis magnetic flux density distribution of each fluxgate magnetic sensor at this position is determined. In this way, we can obtain the 3-axis magnetic field data of the 8-fluxgate sensor at 101 equally spaced positions between 1000 mm and 4000 mm. The interpolation method is depicted in Figure 8.

**Figure 9:**
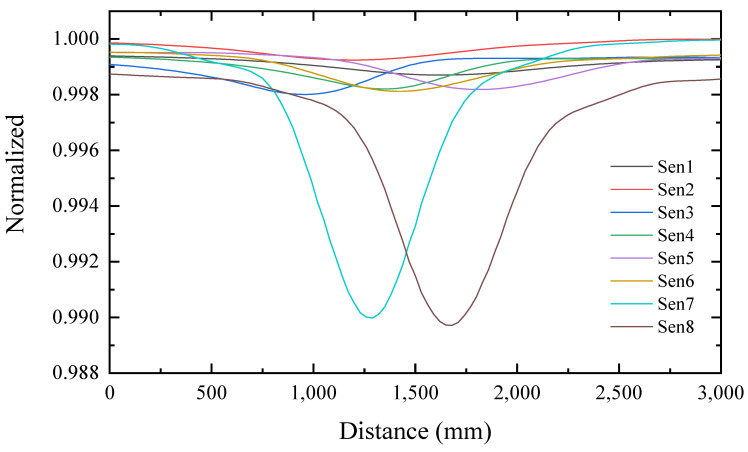
Normalized total field distribution plot.

According to the above processing method, we process each set of data into a set of magnetic flux tensor matrices. The resulting matrix is mapped to the labels one-to-one. The total field distribution for the entire survey line is shown in Figure 9. From Figure 9, it can be found that the amplitude of the signal received by the No. 7 and No. 8 sensors is the largest because these two sensors are closest to the target. The distance between the two valleys is approximately 400 mm, which is the distance between the two sensors.

### 3.2. Uncertainty Analysis

The Earth’s magnetic field is a dynamic system and varies on a wide range of timescales from seconds to hundreds of millions of years. The core field [37] is dominated by fields generated from a self-sustaining dynamo in the Earth’s fluid outer core. This creates around 95% of the magnetic field strength at the Earth’s surface. The measured magnetic field averages a strength on the order of 50 μT, varying, however, between some 20 and 60 μT. Another internal source is the lithospheric field, generated in rocks containing minerals carrying the magnetization and situated below the Curie temperature, generally in the upper 5–30 km of the Earth’s surface [38]. Globally, this contribution is much smaller at around 20 nT. Regular changes in solar activity and corresponding outer space plasma flow alterations induce global geophysical field fluctuations with a frequency range from 0.001 Hz to 10 Hz [39].

The experimenter pulled the FMCAS by the traction rope to complete the magnetic field measurement of a survey line on the slide rail, and it took about 10s to slide once. Uncertainty in the data collected by the sensors was due to fluctuations in the geomagnetic field. The fluxgate sensor was left standing and collected to record the local magnetic field environment. The sampling frequency was set to 100 Hz and the acquisition time was 30 s. The fluxgate sensor recorded data on the variation in the three components of the environmental magnetic field. The data were statistically observed for the uncertainty of the magnetic field, as shown in Table 4.

**Figure 10:**
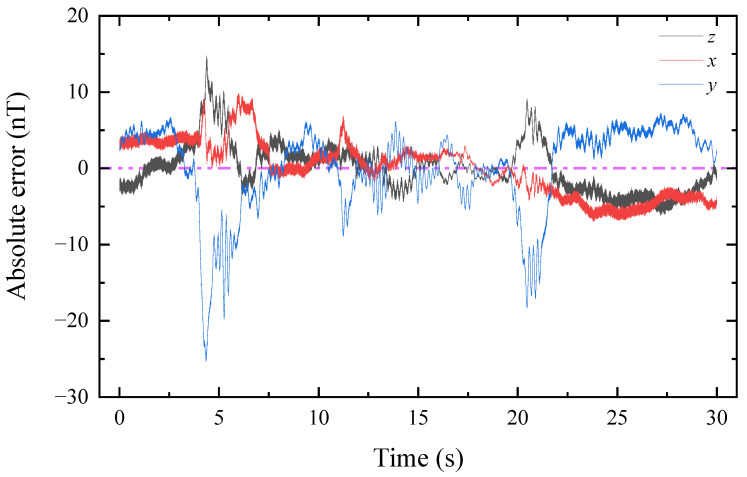
Absolute error diagram of ambient magnetic field in three axes.

From Table 4, we see that the ambient magnetic field fluctuates a lot, and the uncertainty of the collected data is caused by the fluctuation of the ambient magnetic field. We subtracted the mean value from the measured triaxial magnetic field to obtain the absolute error image of the triaxial magnetic field, as shown in Figure 10.

**Figure 11:**
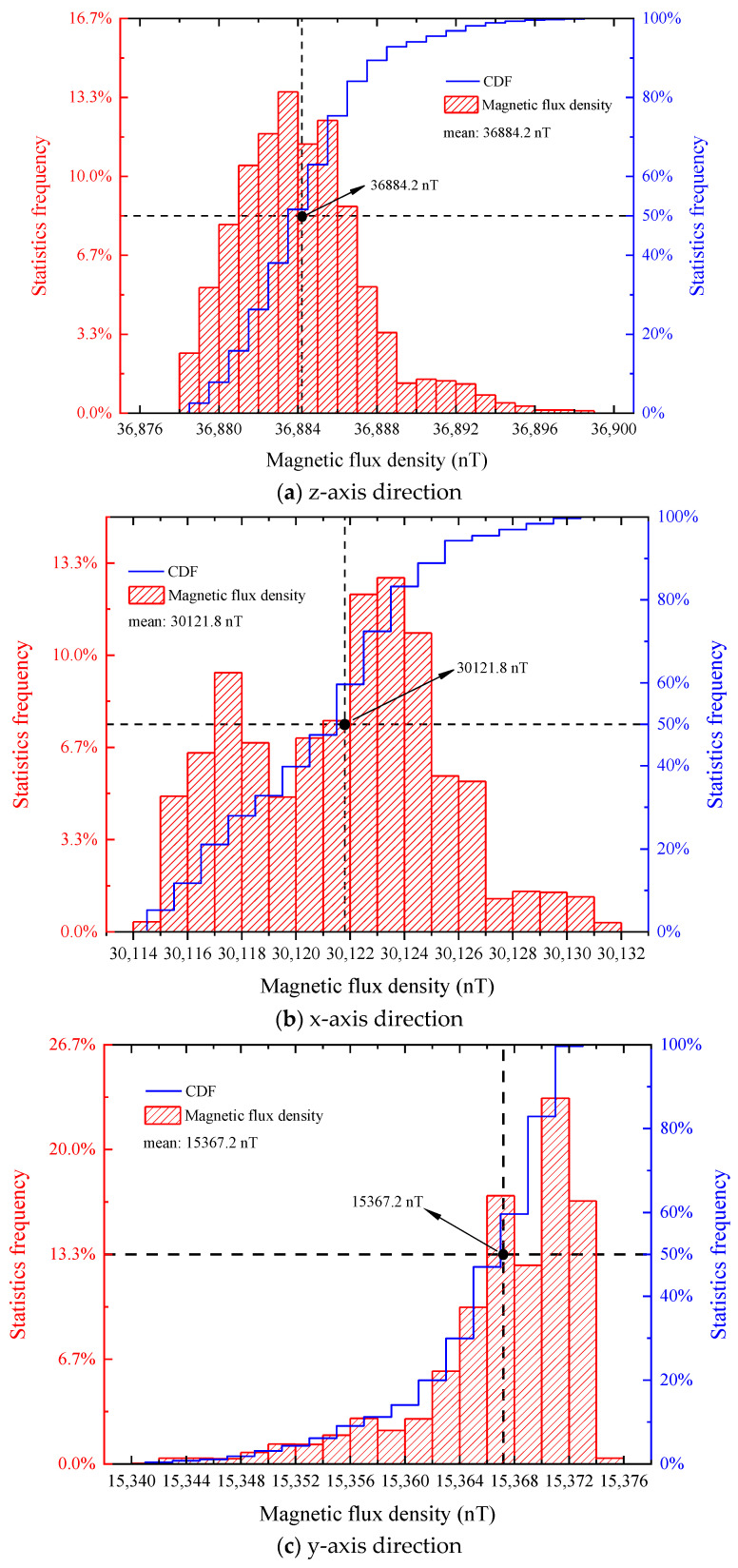
Statistical distribution graph.

By observing Figure 10, we could find that the fluctuation of the magnetic field has a random nature, and the fluctuation of the magnetic field in the x-direction is greater than that in the z- and x-directions. In order to observe the random fluctuation of the ambient magnetic field more intuitively and accurately, we made a statistical distribution chart of the three-axis ambient magnetic field, as shown in Figure 11.

Figure 11 shows that the CDF curves of the z-axis and x-axis components of the environment magnetic field are approximately normally distributed. Z-axis environment magnetic field components are concentrated around the mean value of 36,884.2 nT, and the CDF at 0.5 takes the value of 36,883.5 nT, which is close to the mean value. X-axis environment magnetic field components are concentrated around the mean value of 30,121.8 nT, and the CDF at 0.5 takes the value of 30,121.5 nT, which is close to the mean value. The CDF curve of the y-axis ambient magnetic field component is still a bit different from the normal distribution. The y-axis ambient magnetic field component is not obviously concentrated around the mean value of 15,367.2 nT, but is closer to 15,371 nT, but the CDF value at 0.5 is close to the mean value.

**Figure 12:**
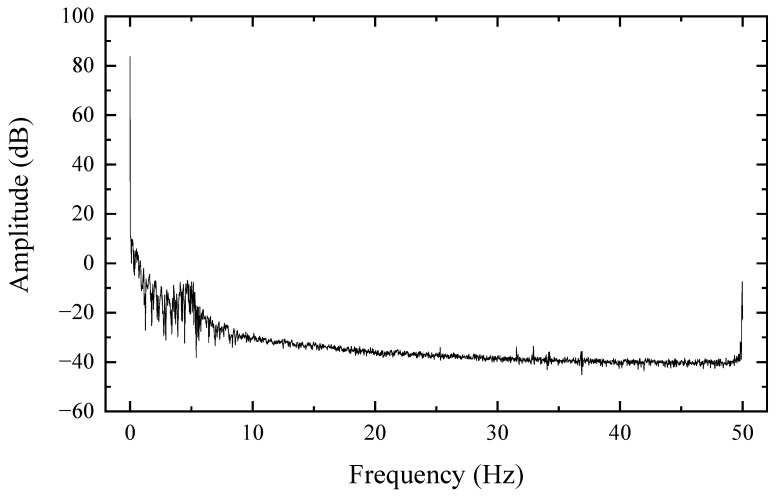
Fast Fourier expansion of the environmental magnetic field in the z-axis direction.

In general, the z-axis and x-axis components of the environment magnetic field are relatively stable. The z-axis direction ambient magnetic field fluctuates approximately 20 nT every 30 s, the x-axis direction ambient magnetic field fluctuates approximately 17 nT every 30 s, and the y-axis direction ambient magnetic field fluctuates approximately 33 nT every 30 s. We observe the frequency distribution of the environment magnetic field based on the collected time-domain signal by fast Fourier transform, as shown in Figure 12.

Observing Figure 12, we can find that the noise is mainly concentrated at low frequencies and 50 Hz. In order to reduce the error of these uncertainties on the data, we use low-pass filtering for the data. As shown in Figure 7, it can be seen that there are obvious deviations between the unfiltered data and the low-pass filtered data, and the two curves do not overlap; these deviations are mainly caused by the 50 Hz noise. According to the experimental site view, there are interference objects such as transmission lines around the environment.

### 3.3. Training the Recognition Network

**Figure 13:**
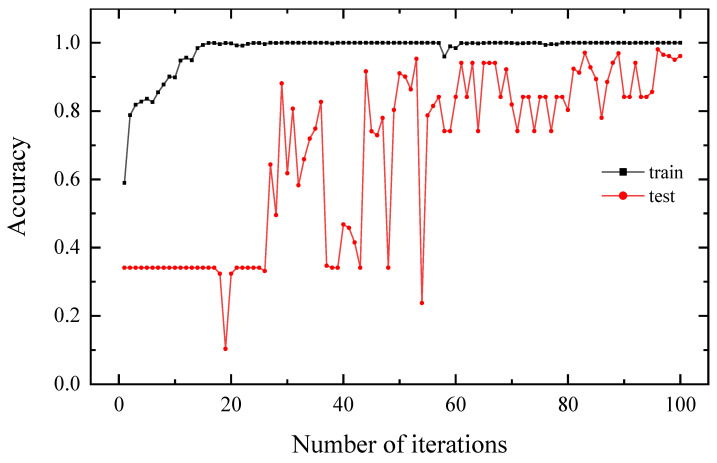
The curve of iterative convergence. The accuracy on the training set is shown by the black line, while the accuracy on the test set is represented by the red line.

We separated the dataset into 3088 training sets, 513 test sets, and 100 validation sets, according to Table 2. The three categories are mutually exclusive and fully distinct. The model training required four hours after 100 iterations, as depicted in Figure 13.

For sample set D, the classification accuracy is defined by the following formula.

(3) $$\mathrm{acc}(f;D)=\frac{1}{m}\sum_{i=1}^{m}Ⅱ\left(f(x_{i})=y_{i}\right)$$

The *f* function represents the completely trained neural network, and m indicates the total number of all samples. The accuracy of the training set steadily increases as the number of iterations grows and eventually converges to 1; the accuracy of the test set gradually increases after the 60th iteration, eventually converging to approximately 0.9. We add seven sets of untargeted ambient magnetic fields to the validation set, totaling 107 groups, to further verify the recognition effect. Table 5 shows the test labels in the final validation set.

We incorporate an untargeted environmental magnetic field to solve the accurate target recognition problem. We set the threshold for target recognition to 0.75. If, during the recognition process, it is inferred that the recognition target has a probability greater than 75% and is identified as a target in the target library, we consider this target recognition to be successful. If it is deduced that the recognition probability of the identified target is 75%, we conclude that the target we detected is not in the target library and that it is no see. Including the validation set in the model increased its accuracy by 84.1 percent. The accuracy rate represents the correct proportion of class predictions in the sample. The precision rate represents the proportion of true positive examples in the samples that are predicted to be positive examples. The recall rate represents the proportion of real positive samples that are predicted to be positive samples. F1-Score is a model evaluation index that takes into account both the precision rate and the recall rate. F1-Score is actually the harmonic mean of the precision rate and the recall rate. The recall rate, accuracy rate, precision rate, specificity rate, and F1-Score value are used as performance metrics to evaluate the model.

(4) $$Accuracy=\frac{TP+TN}{TP+FP+TN+FN}$$

(5) $$Recall=\frac{TP}{TP+FN}$$

(6) $$Precision=\frac{TP}{TP+FP}$$

(7) $$Specificity=\frac{TN}{TN+FP}$$

(8) $$F1=\frac{2\times Recall\times Precision\;}{\;Recall+Precision\;}$$

**Figure 14:**
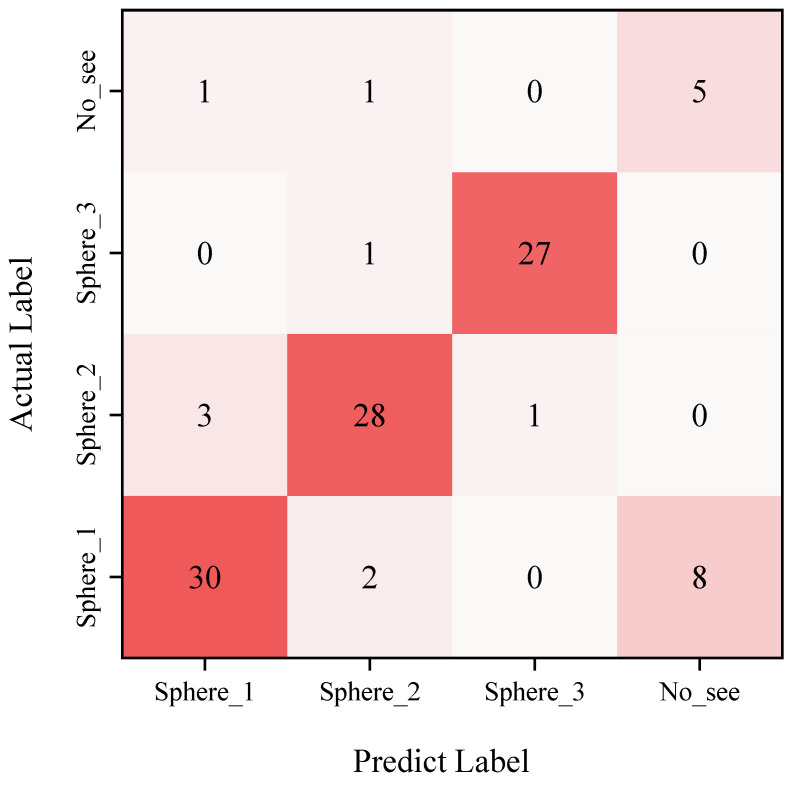
Confusion Matrix.

**Figure 15:**
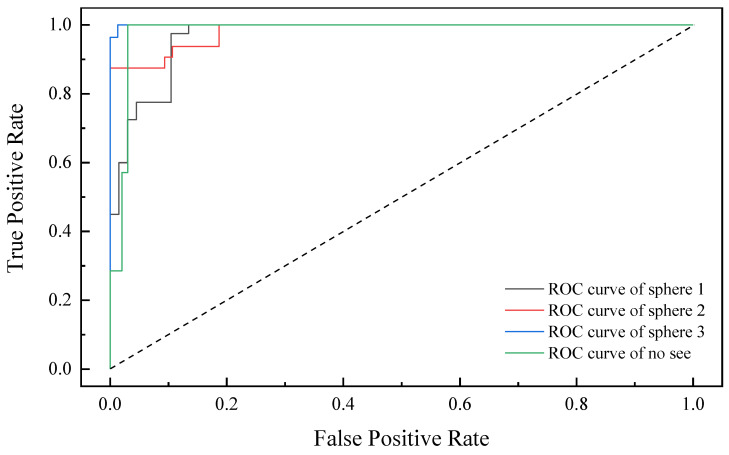
Receiver operating characteristic classification diagram.

TP denotes a true positive case, FP denotes a false positive example, and FN denotes a false negative example. Figure 14 depicts the confusion matrix derived by calculation. As illustrated in Figure 15, we simultaneously generated the receiver operating characteristics (ROC) curve based on the prediction findings.

The model’s accuracy rating is 84.1% after calculations. Table 6 shows the precision rate, recall rate, single accuracy rate, and F1-Score value.

## 4. Experimental Results and Analysis

According to Equation (1), it can be found that the magnetic field generated by the dipole target decays with the third power of distance. Therefore, when the target is far away, the magnetization field of the detection target cannot be obtained by the fluxgate sensor. We used the model HSF923-2H5-AA fluxgate sensor with the sensor index shown in Table 1, but it did not achieve the expected accuracy during the experiment. To obtain a better signal-to-noise ratio, either the magnitude of the target magnetic moment should be increased or the measurement point should be placed close to the detection target. To ensure the availability of Equation (1) (detection distance is much larger than the target diameter), the distance from the detection location to the target cannot be too close either.

The fluctuation of the ambient magnetic field has a great influence on the experiment. By observing Figure 10 and Table 4, it can be found that the uncertainty of the magnetic field fluctuation in the experimental environment is great, especially the fluctuation in the y-axis direction, and the root mean square of the magnetic field is as high as 5.7 nT within the 30 s of the test, which is much higher than the root mean square of the magnetic field in the x-axis and z-axis directions. Observing Figure 12, it can be found that the noise of the ambient magnetic field is mainly concentrated in the low-frequency and 50 Hz parts, and a better waveform can be obtained by low-pass filtering, as shown in Figure 7. It is recommended to perform a time-frequency analysis of the ambient magnetic field before the experiment to obtain the noise distribution and select a suitable filter to restore the real waveform.

In the experiment, when the No. 1 iron ball target is 0.7 m away from the fluxgate sensor, the magnetic field is only 3 nT. When the ambient magnetic field fluctuates widely, the signal of the target is completely mixed into the ambient magnetic field. When the No. 1 iron ball target is 0.3 m away from the fluxgate sensor, a better signal-to-noise ratio is obtained, and the magnetic field is 120 nT. Figure 13 reveals that the accuracy of the validation set begins to converge after 60 iterations, and the accuracy of the validation set tends to fluctuate smoothly after 80 iterations and then converges at approximately 0.9.

Through the analysis of the recall rate, accuracy rate, and F1 value calculated by the model, it can be seen that in the process of target classification and recognition, the single recognition accuracy of the No. 3 iron ball is the highest, and the F1 index is closest to 1. The identification between the No. 1 iron ball and no see is relatively difficult because the local geomagnetic environment has magnetic anomalies, and the diameter of the No. 3 iron ball is relatively small, which is not conducive to distinguishing the difference between the target and the environment. In other words, the magnetic anomaly caused by the No. 1 iron ball is sometimes overwhelmed by the local environment, so the recognition algorithm cannot accurately distinguish between the two. Similarly, the recognition algorithm also confuses some No. 2 iron balls with No. 1 iron balls because the magnetic anomalies between the two are not very different. In contrast, the algorithm is more accurate in distinguishing No. 1 iron balls and No. 3 iron balls.

The experimental results show that the system can still identify targets with significant discrepancies in the magnetic anomalies.

## 5. Conclusions

When distinguishing metal targets through this recognition algorithm, the overall accuracy rate is 84.1%. The No. 3 iron ball performed the best in recognition, with a single recognition accuracy rate of 96.3%, a recall rate of 96.4%, and a precision rate of 96.4%. The excellent performance is attributed to the fact that the target is larger than other targets, and the magnetic anomaly is more obvious. The fluctuation of the ambient magnetic field leads to increased uncertainty in the whole identification system. In the experimental environment, the main noise is low-frequency and 50 Hz noise. Therefore, a better real waveform can be restored by the low-pass filter. Using the noise-reduced waveform for training recognition will make the neural network converge better. When the algorithm recognizes the target with a relatively close volume, it is accompanied by the generation of errors. In the experiment, the No. 1 iron ball and the No. 2 iron ball were misclassified because the volumes of the two are relatively close, and the magnetic anomalies displayed on the fluxgate magnetic field sensor are relatively close.

Based on the observation of experimental effects, we found that improving the signal-to-noise ratio of the target can classify the target more accurately. For example, during the experiment, the slide rail can be made closer to the target. The rules to be followed throughout the experiment are that the slide is located directly north of the target and the slide is set along the east–west direction. The slide cannot be set to any other position. If the slide is located due south of the target (the slide is still set in the east–west direction), the peaks and valleys of the magnetic anomaly signal are not consistent with the trained model. Such data cannot be identified. Our experimental site is in the northern hemisphere, and if the experiment is conducted in the southern hemisphere, it is better to set the slide rail to the south side of the target (the slide rail is still set in the east–west direction).

In general, our enhanced RestNet-18 network can accurately classify metallic items.

## Figures and Tables

**Table 1:** The performance parameters of the fluxgate.

Performance Name	Range	Bandwidth	Noise
Parameter	−100 uT∼+100 uT	DC∼30 Hz	≤15 pT@1 Hz

**Table 2:** ResNet architectures with different layers.

Layer Name	Output Size	18-Layer	50-Layer
conv1	112 × 112	7 × 7, 64, stride 2	
conv2_x	56 × 56	3 × 3 max pool, stride 2	
		$\left[\begin{array}{c} 3\times 3,\;64\\ 3\times 3,\;64 \end{array}\right]\times 2$	$\left[\begin{array}{c} 1\times 1,\;64\\ \begin{array}{l} 3\times 3,\;64\\ 1\times 1,\;256 \end{array} \end{array}\right]\times 2$
conv3_x	28 × 28	$\left[\begin{array}{c} 3\times 3,\;128\\ 3\times 3,\;128 \end{array}\right]\times 2$	$\left[\begin{array}{c} 1\times 1,\;128\\ \begin{array}{l} 3\times 3,\;128\\ 1\times 1,\;512 \end{array} \end{array}\right]\times 4$
conv4_x	14 × 14	$\left[\begin{array}{c} 3\times 3,\;256\\ 3\times 3,\;256 \end{array}\right]\times 2$	$\left[\begin{array}{c} 1\times 1,\;256\\ \begin{array}{l} 3\times 3,\;256\\ 1\times 1,\;1024 \end{array} \end{array}\right]\times 6$
conv5_x	7 × 7	$\left[\begin{array}{c} 3\times 3,\;512\\ 3\times 3,\;512 \end{array}\right]\times 2$	$\left[\begin{array}{c} 1\times 1,\;512\\ \begin{array}{l} 3\times 3,\;512\\ 1\times 1,\;2048 \end{array} \end{array}\right]\times 3$
1 × 1		average pool, 1000-d fc, softmax	

**Table 3:** Statistical table of measured data.

Detection Target	Properties (Radius/mm)	Valid Data (Groups)	Invalid Data (Groups)
No. 1 iron ball	51	1238	0
No. 2 iron ball	56.5	1223	0
No. 3 iron ball	67.5	1240	1

**Table 4:** Statistical table of environmental magnetic field changes.

Direction	Mean	Standard Deviation	Maximum	Minimum
z	36,884.2	3.3	36,898.9	36,878.0
x	30,121.8	3.6	30,131.6	30,114.7
y	15,367.2	5.7	15,374.4	15,341.8

Unit: nT.

**Table 5:** Validation set contains label table.

Detection Target	Valid Data (Groups)
No. 1 iron ball	40
No. 2 iron ball	32
No. 3 iron ball	28
No see	7

**Table 6:** Model reference index table.

	Precision	Recall	Acc Single	F1-Score
No. 1 iron ball	88.2%	75%	86.9%	0.81
No. 2 iron ball	87.5%	87.5%	92.5%	0.87
No. 3 iron ball	96.4%	96.4%	96.3%	0.96
No see	38.5%	71.4%	90.6%	0.5

## Nomenclature

*m*, the magnetic moment of the dipole; *B*_dipolar_, magnetic field produced by a magnetic dipole; *r*, the distance between the magnetic dipole’s center and the observation point; $\hat{r}$, the unit vector between the magnetic dipole’s center and the observation point; *xyz*, the pseudo total field of the fluxgate magnetometer; *x*, the fluxgate magnetometer’s x-axis magnetic field output; *y*, the fluxgate magnetometer’s y-axis magnetic field output; *z*, the fluxgate magnetometer’s z-axis magnetic field output; G, the magnetic field to obtain the magnetic gradient tensor.

## Greek Symbols

*μ*_0_, the vacuum permeability; π, the ratio of a circle’s circumference to its diameter.

## Subscripts

*i*, fluxgate marking number; z, the direction.

## Glossary

FMCAS, fluxgate magnetometer cube arrangement structure; MCMC, Markov chain Monte Carlo; RCNN, region convolutional neural network; ResNet, residual neural network; CDF, cumulative distribution function; ACC, accuracy; FAC, field aligned currents; UXO, unexploded ordnance.
